# Diagnostic Value of Multiple Serum Biomarkers for Vancomycin-Induced Kidney Injury

**DOI:** 10.3390/jcm10215005

**Published:** 2021-10-27

**Authors:** Sang-Mi Kim, Hyun-Seung Lee, Min-Ji Kim, Hyung-Doo Park, Soo-Youn Lee

**Affiliations:** 1 Samsung Medical Center, Department of Laboratory Medicine and Genetics, School of Medicine, Sungkyunkwan University, Seoul 06351, Korea; jeehee0520@gmail.com (S.-M.K.); hyunseung1011.lee@samsung.com (H.-S.L.); nayadoo@hanmail.net (H.-D.P.); 2 Biomedical Statistics Center, Research Institute for Future Medicine, Samsung Medical Center, Seoul 06351, Korea; rabbit93.kim@samsung.com; 3 Samsung Medical Center, Department of Clinical Pharmacology & Therapeutics, School of Medicine, Sungkyunkwan University, Seoul 06351, Korea; 4 Department of Health Science and Technology, Samsung Advanced Institute of Health Science and Technology, Sungkyunkwan University, Seoul 06351, Korea

**Keywords:** vancomycin-induced kidney injury, vancomycin, nephrotoxicity, serum biomarker, trefoil factor-3, cystatin C, tumor necrosis factor receptor 1, osteopontin

## Abstract

Acute kidney injury (AKI) is a major contributor to in-hospital morbidity and mortality. Vancomycin, one of the most commonly used antibiotics in a clinical setting, is associated with AKI, with its incidence ranging up to 43%. Despite the high demand, few studies have investigated serum biomarkers to detect vancomycin-induced kidney injury (VIKI). Here, we evaluated the diagnostic value of nine candidate serum biomarkers for VIKI. A total of 23,182 cases referred for vancomycin concentration measurement from January 2018 to December 2019 were screened and 28 subjects with confirmed VIKI were enrolled (VIKI group). Age- and sex- matched control group consisted of 21 subjects who underwent vancomycin therapy without developing VIKI (non-VIKI group), and 23 healthy controls (HC group). The serum concentrations of clusterin, retinol binding protein 4 (RBP4), interleukin-18 (IL-18), tumor necrosis factor receptor 1 (TNF-R1), C-X-C motif chemokine ligand 10 (CXCL10), neutrophil gelatinase-associated lipocalin (NGAL), osteopontin, trefoil factor-3 (TFF3), and cystatin C were compared among the three groups, and their correlations with estimated glomerular filtration rate (eGFR) and diagnostic values for VIKI were assessed. All of the biomarkers except clusterin and RBP4 exhibited significant elevation in the VIKI group. Serum TFF3, cystatin C, TNF-R1, and osteopontin demonstrated an excellent diagnostic value for VIKI (TFF3, area under the curve (AUC) 0.932; cystatin C, AUC 0.917; TNF-R1, AUC 0.866; osteopontin, AUC 0.787); and except osteopontin, a strong negative correlation with eGFR (TFF3, r = −0.71; cystatin C, r = −0.70; TNF-R1, r = −0.60). IL-18, CXCL10, and NGAL showed weak correlation with eGFR and moderate diagnostic value for VIKI. This study tested multiple serum biomarkers for VIKI and showed that serum TFF3, cystatin C, TNF-R1, and osteopontin could efficiently discriminate VIKI patients. Further studies are warranted to clarify the diagnostic value of these biomarkers in VIKI.

## 1. Introduction

As the first treatment option for methicillin-resistant *Staphylococcus aureus* (MRSA) infection [1], vancomycin is a commonly used antimicrobial agent whose use has been increasing with the growing prevalence of MRSA infection [2,3]. Although vancomycin plays a critical role, its use is difficult due to the potential for nephrotoxicity and its narrow therapeutic range [4]. Vancomycin-induced kidney injury (VIKI) is reversible with timely discontinuation of the drug or appropriate dose adjustment [5,6]. Early and accurate biomarkers for VIKI could lead to better outcomes in vancomycin-treated patients. Traditionally, guidelines have recommended dose adjustments based on vancomycin concentration and serum creatinine (sCr) [7,8]. However, elevation in sCr concentration is affected by non-renal factors independent of kidney function [9,10] and lags several days behind actual change in kidney function [11]. Moreover, it reflects the downstream effect from a reduction in glomerular filtration rate (GFR) rather than an indicator of VIKI itself.

Several studies have investigated urinary biomarkers for VIKI, such as kidney injury molecule-1 (KIM-1), clusterin, neutrophil gelatinase-associated lipocalin (NGAL), osteopontin, cystatin C, and beta-2 micro-globulin [12,13,14,15,16]. Studies have focused on urinary biomarkers rather than their serum counterparts [17]. However, urinary biomarker concentration can be influenced by urine concentration and urinary flow rate depending on patient hydration status or administration of diuretics. On the contrary, serum biomarkers are less susceptible to bacterial infection, and obtainable even in oliguric/anuric patients [18]. However, except our previously conducted serum NGAL study, no other studies have been performed on serum biomarkers for VIKI [18]. In that study, we evaluated serum NGAL as a renal function marker in vancomycin-treated patients. However, it demonstrated limited value for monitoring renal function in patients with high blood leukocyte count. Serum cystatin C, a cysteine proteinase inhibitor protein [19], is excreted through glomerular filtration and tubular secretion [20,21]. It is a widely investigated glomerular filtration marker and has been evaluated as a predictor for vancomycin clearance in a number of studies [22,23,24,25,26,27,28,29,30]. However, its value as an indicator of VIKI has yet to be evaluated.

In this present study, we evaluated the value of serum concentrations of biomarkers that have been identified as potential urinary biomarkers for VIKI (osteopontin, NGAL, clusterin) and other drug-induced kidney injury (trefoil factor-3 (TFF3)) for diagnosis of VIKI [31,32,33]. Despite more than 60 years of clinical use, the pathophysiology of VIKI has not been elucidated due to the cost of clinical trials [12,34]. Based on experimental animal model studies, VIKI is suggested to be associated with oxidative stress caused by reabsorbed vancomycin accumulated in the lysosomes of proximal tubular cells [12,35,36]. Therefore, the biomarkers shown to reflect tubular injury in previous publications were also evaluated, such as cystatin C, tumor necrosis factor receptor 1 (TNF-R1), interleukin-18 (IL-18), C-X-C motif chemokine ligand 10 (CXCL10), osteopontin, and retinol binding protein 4 (RBP4) [37,38,39]. Here, we aim to identify candidate serum biomarkers for the diagnosis of VIKI.

## 2. Results

### 2.1. Subject Characteristics

A total of 72 subjects was included; median age was 61 years (interquartile range [IQR], 53–67 years). No significant difference was observed in age or sex among the three groups (Table 1). The sCr level was higher in the VIKI group compared with non-VIKI and healthy control (HC) groups; however, estimated GFR (eGFR) was lower in the VIKI group (VIKI vs. non-VIKI vs. HC, 42 mL/min/1.73 m^2^ vs. 107 mL/min/1.73 m^2^ vs. 85 mL/min/1.73 m^2^, *p* < 0.001). Vancomycin trough concentration (measured within 24 h before or after sCr measurement) was significantly higher in the VIKI group than in the non-VIKI group (VIKI vs. non-VIKI, 27.3 ± 5.3 µg/dL vs. 14.3 ± 2.8 µg/dL, *p* < 0.001). C-reactive protein (CRP) was higher in the VIKI group and non-VIKI groups compared with the HC group (VIKI vs. non-VIKI vs. HC, 5.78 mg/dL vs. 6.31 mg/dL vs. 0.04 mg/dL, *p* < 0.001), but was similar between the VIKI and non-VIKI groups (*p* = 0.955). The most common indication for vancomycin use was respiratory infection both in the VIKI (9/28, 32%) and non-VIKI (7/21, 33%) group. Baseline characteristics of the 55 subjects enrolled for the screening test were similar (data not shown).

### 2.2. Comparison of Serum Biomarker Concentrations

**Figure 1:**
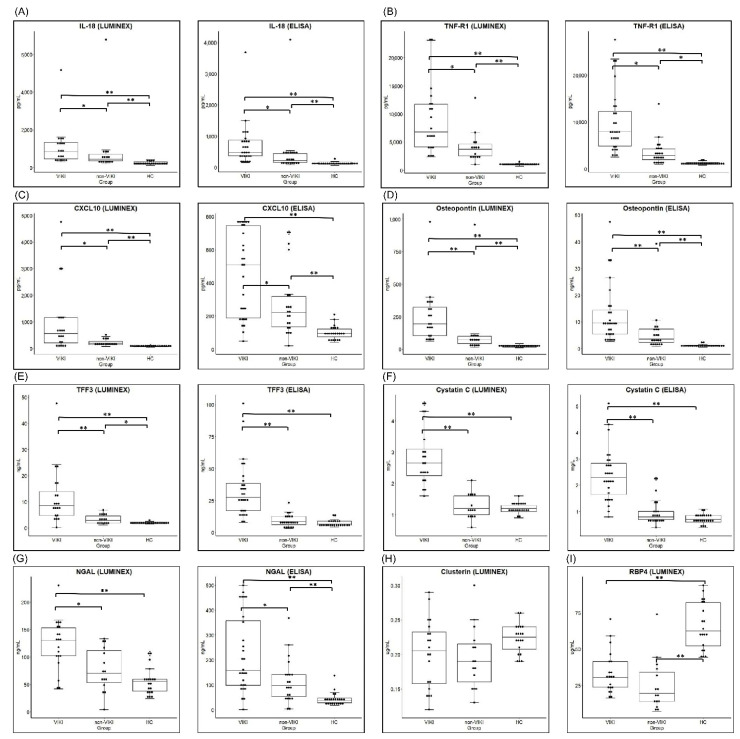
Comparison of serum biomarker concentration between the VIKI, non-VIKI and HC group. Box and whisker plot of serum concentration of (**A**) IL-18 (**B**) TNF-R1 (**C**) CXCL10, (**D**) osteopontin, (**E**) TFF3, (**F**) cystatin C, (**G**) NGAL, (**H**) clusterin, and (**I**) RBP4 measured in the screening test by Luminex assay and by enzyme-linked immunosorbent assay (ELISA). For each box, horizontal lines inside the box represent the interquartile range and the median, respectively and the whiskers represents the 10th and 90th percentiles. Each dot represents the individual values. * *p* value < 0.05, and ** *p* value < 0.001, respectively. Abbreviations: VIKI, vancomycin-induced kidney injury; HC, healthy control; IL-18, interleukin-18; TNF-R1, tumor necrosis factor receptor 1; CXCL10, C-X-C motif chemokine ligand 10; TFF3, trefoil factor-3; NGAL, neutrophil gelatinase-associated lipocalin; RBP4, retinol binding protein 4.

The comparison results of serum biomarker concentrations among the three groups are summarized in Figure 1 and Table 2. In the screening test (Figure 1 and Table 2A) performed using the Luminex assay, all biomarkers except clusterin and RBP4 showed significant difference between the VIKI and control (non-VIKI and HC) group. In the comparison among the three groups, clusterin showed no significant difference and RBP4 failed to show difference between the VIKI and non-VIKI groups in post-hoc comparison.

Among the nine biomarkers, the seven (IL-18, TNF-R1, osteopontin, TFF3, cystatin C, CXCL 10, and NGAL) that showed significant difference between the VIKI and control (non-VIKI and HC) groups in the screening test were included for quantification using ELISA. Serum concentration of all seven biomarkers showed significant difference in the comparison among the three groups (Figure 1 and Table 2B). In post-hoc comparison, the serum concentration of TFF3 was significantly higher in the VIKI group compared with the HC or non-VIKI group but without significant difference between the HC and non-VIKI groups (VIKI vs. non-VIKI vs. HC, 27.5 (16.8–39.0) ng/mL vs. 8.4 (6.1–12.4) ng/mL vs. 6.5 (5.7–8.9) ng/mL, *p* < 0.001). Similarly, serum cystatin C concentration was significantly elevated in the VIKI group compared with the HC or non-VIKI group but without significant difference between the HC and non-VIKI groups (VIKI vs. non-VIKI vs. HC, 2.3 (1.6–2.8) mg/L vs. 0.8 (0.7–1.0) mg/L vs. 0.7 (0.6–0.9) mg/L, *p* < 0.001). Serum IL-18, TNF-R1, CXCL10, osteopontin, and NGAL concentrations were significantly higher in the VIKI group compared with the HC and non-VIKI groups, and they also showed significant difference between the non-VIKI and HC groups.

### 2.3. Correlation between the Serum Biomarker Concentration and eGFR

**Figure 2:**
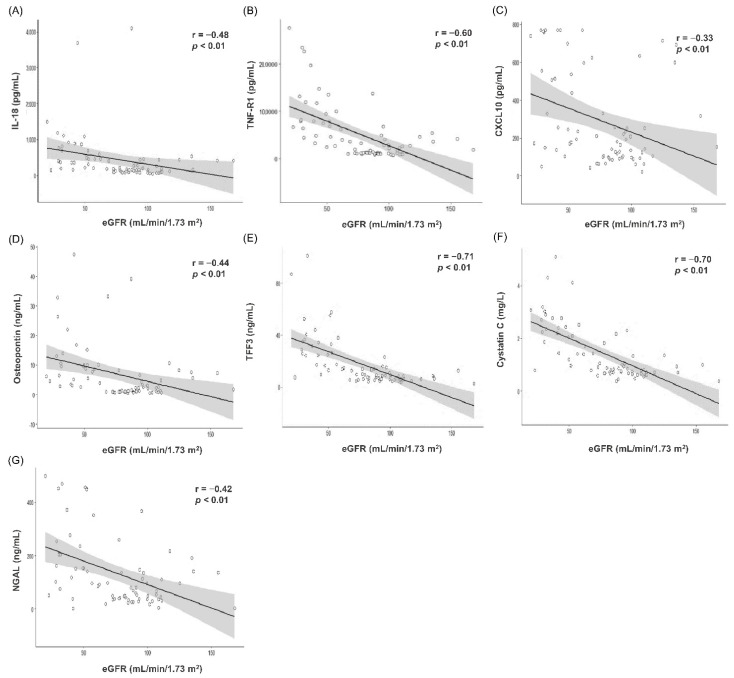
Correlation analyses and scatter plots between serum biomarker concentrations measured by enzyme-linked immunosorbent assay (ELISA) and estimated glomerular filtration rate (*n* = 72). (**A**) IL-18 (**B**) TNF-R1 (**C**) CXCL10 (**D**) Osteopontin (**E**) TFF3 (**F**) Cystatin C (**G**) NGAL. In the scatter plot, the solid line and gray zone represent the regression line and its 95% confidence interval, respectively. The value of r represents the correlation coefficient by Spearman’s rho method. Abbreviation: eGFR, estimated glomerular filtration rate; IL-18, interleukin-18; TNF-R1, tumor necrosis factor receptor 1; CXCL10, C-X-C motif chemokine ligand 10; TFF3, trefoil factor-3; NGAL, neutrophil gelatinase-associated lipocalin.

Scatter plot and Spearman’s correlation analysis results between serum biomarker concentration measured using ELISA and eGFR are summarized in Figure 2. TFF3 (r = −0.71, *p* < 0.001, Figure 2E) and cystatin C (r = −0.70, *p* < 0.001, Figure 2F) showed strong, negative correlation with eGFR, while TNF-R1 (r = −0.60, *p* < 0.001, Figure 2B) showed moderate, negative correlation. IL-18 (r = −0.48, *p* < 0.001, Figure 2A), CXCL10 (r = −0.33, *p* = 0.004, Figure 2C), osteopontin (r = −0.44, *p* < 0.001, Figure 2D), and NGAL (r = −0.42, *p* < 0.001, Figure 2G) showed weak, negative correlation with eGFR.

Consistent results were observed in the screening test (Supplementary Figure S1). Cystatin C (r = −0.84, *p* < 0.001) and showed strong negative correlation with eGFR, while TFF3 (r = −0.64, *p* < 0.001) and TNF-R1 (r = −0.52, *p* < 0.001) showed moderate negative correlation. CXCL10 (r = −0.36, *p* = 0.007), osteopontin (r = −0.47, *p* < 0.001), NGAL (r = −0.44, *p* < 0.001), and IL-18 (r = −0.41, *p* = 0.002) showed weak, negative correlation with eGFR; however, RBP4 and clusterin failed to demonstrate significant correlation with eGFR.

### 2.4. Logistic Regression Analysis

To evaluate the association between each biomarker and the development of VIKI in vancomycin-treated patients (*n* = 49), we preformed univariate and multivariable logistic regression analysis with the concentration of each biomarker and the clinical features that might be potential factors related to VIKI as predictor variables and the development of VIKI as the outcome.

In the univariate logistic regression analysis (Supplementary Table S1), the only independent variables to show a statistically significant association with VIKI were serum TNF-R1 (odds ratio (OR) = 1.000, 95% confidence interval (CI) = 1.000–1.001, *p* = 0.003), CXCL10 (OR = 1.003, 95% CI = 1.001–1.006, *p* = 0.017), TFF3 (OR = 1.000, 95% CI = 1.000–1.000, *p* = 0.002), cystatin C (OR = 1.003, 95% CI = 1.001–1.004, *p* < 0.001) and NGAL (OR = 1.007, 95% CI = 1.001–1.013, *p* = 0.016). Similar results were observed in the multivariable logistic regression model adjusted for age, sex, BMI and CRP (Table 3): serum TNF-R1 (odd ratio (OR) = 1.000, 95% confidence interval (CI) = 1.000–1.001), CXCL10 (OR = 1.003, 95% confidence interval (CI) = 1.000–1.006), osteopontin (OR = 1.000, 95% CI = 1.000–1.000), TFF3 (OR = 1.000, 95% CI = 1.000–1.001), cystatin C (OR = 1.003, 95% CI = 1.001–1.005) and NGAL (OR = 1.007, 95% CI = 1.001–1.013) showed significant associations with the development of VIKI, and might serve as independent predictors for VIKI (all *p* < 0.05).

### 2.5. Diagnostic Performance of Serum Biomarkers for Detecting VIKI

**Figure 3:**
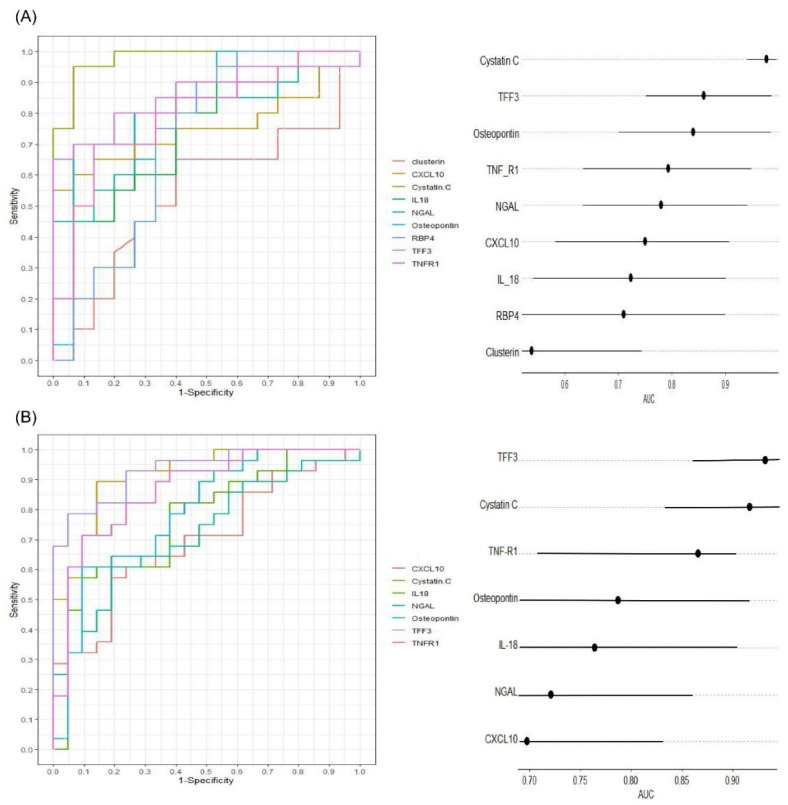
Diagnostic performance of the serum biomarker concentration measured by Luminex assay ((**A**), *n* = 35) and ELISA ((**B**), *n* = 49) for vancomycin-induced kidney injury (VIKI) in subjects receiving vancomycin (non-VIKI and VIKI group). Receiver operating characteristic curves (left) and horizontal dot plot of area under curve (right) are shown. In the horizontal dot plot, the dot and line represent the area under curve and its 95% confidence interval, respectively. Abbreviations: TFF3, trefoil factor-3; IL-18, interleukin-18; TNF-R1, tumor necrosis factor receptor 1; NGAL, neutrophil gelatinase-associated lipocalin; CXCL10, C-X-C motif chemokine ligand 10; RBP4, retinol binding protein 4; AUC, area under curve.

The diagnostic performance of the biomarkers for detecting VIKI was evaluated in vancomycin-treated subjects (VIKI and non-VIKI groups) using the receiver operating characteristic (ROC) analysis. Both in the screening test using Luminex assay (Figure 3A, *n* = 35) and in ELISA (Figure 3B, *n* = 49), cystatin C (area under the curve (AUC) 0.977 in the Luminex assay, 0.917 in the ELISA), TFF3 (AUC 0.860 in the Luminex assay, 0.932 in the ELISA), TNF-R1 (AUC 0.793 in the Luminex assay, 0.866 in the ELISA) and osteopontin (AUC 0.840 in the Luminex assay, 0.787 in the ELISA) showed excellent to outstanding diagnostic value for VIKI. All the biomarkers except clusterin (AUC 0.538 in the Luminex assay) showed acceptable diagnostic value.

## 3. Discussion

VIKI occurs in approximately 5–43% of vancomycin-treated patients [40,41]. With more than 3 million patients estimated to undergo vancomycin treatment annually [42], the attributable damage of VIKI affects about 300,000 people annually in the United States alone [13]. Owing to its reversible nature, a biomarker that could detect VIKI more accurately and rapidly could lead to better outcomes in these patients. Urinary KIM-1, clusterin, NGAL, osteopontin, cystatin C, and beta-2 micro-globulin have been investigated as biomarkers for VIKI [12,13,14,15,16], and studies have shown urinary KIM-1 [13,14,15], clusterin [13], NGAL [15], and osteopontin [13] have this potential. However, serum biomarkers for VIKI have not yet been studied. To the best of our knowledge, no other studies have been performed except our previous study on serum NGAL [43]. Therefore, we aimed to evaluate the diagnostic value of the serum concentrations of biomarkers whose urine concentrations have exhibited promising results for detection of VIKI (osteopontin, NGAL, clusterin) or other drug-induced kidney injury (TFF3) and those that have shown to reflect renal tubular injury (CXCL10, IL-18, TNF-R1, osteopontin, RBP4, and cystatin C) for the diagnosis of VIKI [37,39,44]. In our study, serum TFF3, cystatin C, TNF-R1, and osteopontin showed the best diagnostic value for VIKI.

TFF3 is a small peptide hormone mostly expressed in mucus-producing epithelial cells within the gastrointestinal tract [45]. Its serum concentration increases with inflammation of the gastrointestinal tract [46,47]. In kidneys, it is produced by cells of the collecting ducts [48] and renal tubular epithelial cells [49]. Its physiological function within the kidneys has yet to be elucidated. However, based on its biological effects, it is assumed that TFF3 takes part in renal repair [50,51]. Both urinary and serum TFF3 concentrations have been shown to significantly increase in CKD patients [51,52,53,54]. In studies regarding drug-induced acute kidney injury, urinary TFF3 level has shown discrepant results. In a human study with kidney injury after cisplatin treatment, urinary TFF3 level elevated twofold [55]. However, in a study with rodents, urinary TFF3 concentration decreased in acute renal tubular injury [56]. The cause of this discrepant result between human and rat studies remains uncertain. Yet neither urinary nor serum TFF3 have been studied in VIKI. In our study, serum TFF3 concentration was increased in the VIKI group without a significant increase in the non-VIKI group. Moreover, it exhibited a strong negative correlation with eGFR (r = −0.71) and excellent diagnostic value for VIKI (AUC 0.932). Since the mechanism of VIKI involves renal tubular cells [12,35,36] and the urine concentration of TTF3 has shown an association with acute tubular injury [55,56], serum TFF3 could potentially be used as a biomarker for VIKI. However, further studies with larger populations and serial quantifications of serum TFF3 concentration according to the chronological progression to VIKI onset should be conducted to increase qualification of serum TFF3 as an early and accurate marker of VIKI.

Serum cystatin C is filtered freely by the glomeruli with near-complete reabsorption in the proximal tubule without being affected by muscle mass, diet, or sex. Accordingly, it has been investigated widely as a glomerular filtration marker that could outperform serum creatinine in terms of accuracy [22,23,24,25,26,27,28,29,30,57,58]. It has shown to better predict vancomycin clearance compared to serum creatinine [22,23,24,25,26,27,28,29,30]. Cystatin C is excreted also through tubular secretion [20,21]. Accordingly, urinary cystatin C has been the subject of drug-induced nephrotoxicity [31], including VIKI [13,14,16]. However, the value of serum cystatin C as a biomarker for VIKI has not been widely studied. In our study, serum cystatin C concentration was significantly increased only in the VIKI group among the three groups demonstrating outstanding diagnostic value for VIKI (AUC 0.917). Moreover, it was a significant predictor for VIKI in multivariable logistic regression (OR 1.003, *p* < 0.001). However, it is indistinguishable, from our result, whether this increase reflects reduction in glomerular filtration or renal tubular injury.

TNF-R1 is an inflammatory mediator expressed in almost all cell types [59]. In kidneys, it is expressed in the glomerular endothelium, peritubular capillary endothelial cells [60], and distal tubule cells [61]. Elevation in serum TNF-R1 concentration has exhibited a strong association with increased risk of end-stage renal disease in type 2 diabetes [62]. However, it is also elevated in sepsis and autoimmune disorders [63]. Neither urinary nor serum TNF-R1 has been studied in AKI including VIKI. In our study, although serum TNF-R1 concentration was elevated in the non-VIKI group compared with HC, the extent of elevation in the VIKI group was prominent compared with that in the non-VIKI group and showed excellent diagnostic value for VIKI (AUC 0.793 in the Luminex assay, 0.866 in the ELISA) in vancomycin-treated subjects, implying its potential as a biomarker of VIKI.

Osteopontin is an extracellular matrix protein ubiquitously expressed in main organs and apparatuses [64], intervening in various cell activities including cell proliferation and inflammatory responses [65]. Found in the thick ascending limbs of the loop of Henle and in distal nephrons, several human studies have reported that osteopontin could be a promising biomarker for various kidney diseases including urolithiasis, acute and chronic kidney diseases, and renal allograft dysfunction [38]. For VIKI, urinary osteopontin concentrations have been shown to reflect the extent of kidney damage and vancomycin exposure in rat model [13,16]. However, serum osteopontin has not been investigated in VIKI. In our study, serum osteopontin concentration exhibited similar results to TNF-R1, showing a significant difference among three groups with prominent elevation in the VIKI group and excellent diagnostic value for VIKI (AUC 0.840 in the Luminex assay, 0.787 in the ELISA). However, it demonstrated a weak correlation with eGFR (r = −0.44, *p* < 0.01), implying a lack of association with glomerular filtration.

Serum TFF3, cystatin C, TNF-R1 and osteopontin were shown to be expressed in renal tubular cells or in distal nephrons in previous animal model studies. Considering our study results, these serum biomarkers could have potential use in future biomarker studies on VIKI.

The concentration of serum IL-18, TNF-R1, CXCL10, osteopontin, NGAL and RBP4 were significantly different between the non-VIKI and HC groups. Making assumptions from their well-elucidated biological roles, the change of their concentration in the non-VIKI group could be attributable to either inflammatory status of the patient or the effects of vancomycin on the kidney.

There are some limitations that must be noted. First, owing to the retrospective nature of our study, the study evaluated the diagnostic value of the biomarkers rather than their predictive value for VIKI. Secondly, this study identified candidate serum biomarkers that have potential for the diagnosis of VIKI, but the current design could not show their specificity for VIKI. Indeed, we are conducting an additional experiment with a control group consisting of patients with AKI induced by causes other than vancomycin to identify the specificity of the biomarkers for VIKI. Lastly, this research was a single-center study with a limited number of subjects with heterogeneous baseline conditions, preventing our results from being free of bias. Large-scale, multi-center studies would be required to establish the credibility of our findings.

Despite these limitations, this is the first study to assess multiple candidate serum biomarkers for VIKI with the aim of evaluating their potential for the diagnosis of VIKI.

Accurate diagnosis of VIKI remains a challenge in clinical practice. Our study has shown serum cystatin C, TFF3, TNF-R1, and osteopontin as potential biomarkers for VIKI. Further large-scale, prospective, multi-center studies are necessary to confirm this result, and serial quantifications are needed to determine whether these biomarkers could detect kidney injuries with sufficient lead-time for preventive strategy.

## 4. Materials and Methods

### 4.1. Study Subjects and Samples

**Figure 4:**
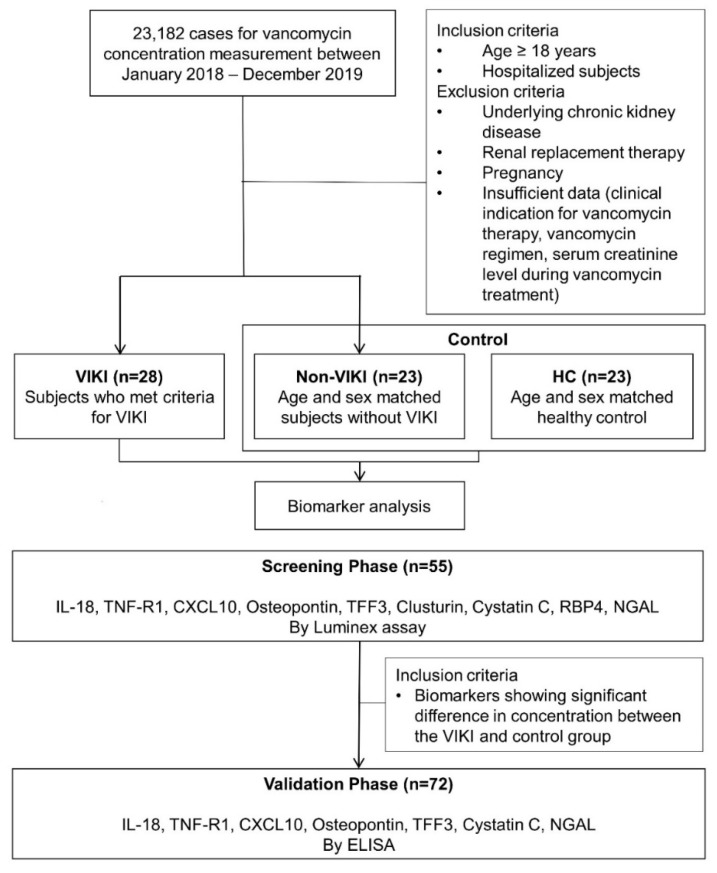
Schematic flow chart of subject selection and serum biomarker analyses. Abbreviations: VIKI, vancomycin-induced kidney injury; HC, healthy control; IL-18, interleukin-18; TNF-R1, tumor necrosis factor receptor 1; CXCL10, C-X-C motif chemokine ligand 10; TFF3, trefoil factor-3; RBP4, retinol binding protein 4; NGAL, neutrophil gelatinase-associated lipocalin; ELISA, enzyme-linked immunosorbent assay.

Study design and subject selection are summarized in Figure 4. The current study included subjects from our previous study cohort [66]. In brief, 23,182 cases referred for vancomycin concentration measurement from January 2018 to December 2019 were screened. Among adult (age ≥ 18 years) hospitalized patients who received intravenous infusion of vancomycin, those who had at least one steady-stage vancomycin trough concentration, and sCr measurement during vancomycin therapy were included. Patients with chronic kidney disease, patients on renal replacement therapy, pregnant patients, and patients with insufficient data (vancomycin regimen, the use and type of concomitant nephrotoxic agent use, clinical indication for vancomycin therapy, sCr during vancomycin therapy) were excluded. Among these patients, those who developed VIKI were included in the VIKI group, and age- and sex- matched patients who did not develop VIKI were included in the non-VIKI group. VIKI was defined as a minimum of two consecutive documented increases in sCr (defined as an increase of 0.3 mg/dL or ≥50% increase from baseline, whichever was greater) after the start of vancomycin therapy and the exclusion of any other possible documented cause for acute kidney injury [8]. The age- and sex- matched control group consisted of two subgroups, the non-VIKI group and healthy control (HC). HC consisted of healthy subjects referred for routine health examination without abnormal medical findings.

This study was approved by the Institutional Review Board at Samsung Medical Center (IRB File No. 2017-12-038). The need for written informed consent was waived due to the retrospective nature of the study.

Clinical samples referred for sCr measurement were collected for serum biomarker measurement. In the VIKI group, clinical samples at the time of VIKI diagnosis were collected; in the non-VIKI group, those drawn within 1 day from the measurement of steady-state vancomycin trough concentration were collected. Peripheral venous blood samples were drawn by venipuncture into a serum separator tube and centrifuged for 10 min at 2270× *g*. The obtained supernatants were stored in an Eppendorf tube at −70 °C until analysis.

### 4.2. Measurement of Serum Biomarker

Schematic workflow of the serum biomarker analyses is also shown in Figure 4. In the screening phase, serum IL-18, TNF-R1, CXCL10, osteopontin, TFF3, clusterin, cystatin C, RBP4, and NGAL concentrations were measured in 55 samples (VIKI group, *n* = 20; non-VIKI group, *n* = 15; HC, *n* = 20). They were analyzed using the Bio-Plex^®^ MAGPIX™ multiplex reader (Bio-Rad Laboratories Inc., Hercules, CA, USA) based on Luminex technology with Human Magnetic Luminex Performance Assay Base Kit, Kidney Biomarker Panel (R&D Systems, Inc., Minneapolis, MN, USA) for CXCL10, osteopontin, TFF3, clusterin, cystatin C, and RBP4 and with the Human Luminex Discovery Assay (R&D Systems, Inc., Minneapolis, MN, USA) for IL-18, TNF-R1, and NGAL. Calibration curves were constructed using Bio-Plex Manager 6.1 (R&D Systems, Inc., Minneapolis, MN, USA).

The seven biomarkers that showed significant difference between the VIKI and control (non-VIKI and HC) groups were selected for further analysis using enzyme-linked immunosorbent assay (ELISA). Serum IL-18, TNF-R1, CXCL10, osteopontin, TFF3, cystatin C, RBP4, and NGAL concentrations were measured in 72 samples (VIKI group, *n* = 28; non-VIKI group, *n* = 21; HC group, *n* = 23) after additional enrollment of 17 subjects (VIKI group, *n* = 8; non-VIKI group, *n* = 6; HC group, *n* = 3). The concentrations were analyzed using the SpectraMax^®^ 190 microplate spectrophotometer (Molecular Devices, LLC, San Jose, CA, USA) based on ELISA using the Human IL-18/IL-1F4 Quantikine ELISA Kit, Human TNF R1/TNFRSF1A Quantikine ELISA Kit, Human CXCL10/IP-10 Quantikine ELISA Kit, Human TFF3 Quantikine ELISA Kit, Human Cystatin C Quantikine ELISA Kit, Human Lipocalin-2/NGAL Quantikine ELISA Kit (R&D Systems, Inc., Minneapolis, MN, USA), and Human Osteopontin (SPP1) ELISA Kit (RayBiotech, Norcross, GA, USA). Calibration curves were constructed using SoftMax Pro 7.0.2 (Molecular Devices, LLC., San Jose, CA, USA). All measurements were performed according to the manufacturer’s instructions.

### 4.3. Clinical Data Collection

Demographic and clinical data including age, sex, body mass index (BMI), sCr, CRP, and comorbidities such as hypertension and diabetes mellitus, and indication for vancomycin use were collected through medical record review. Additionally, therapeutic drug parameters such as duration of vancomycin therapy and concomitant nephrotoxic agents were collected for the VIKI and non-VIKI groups. The eGFR was calculated from sCr using the Chronic Kidney Disease Epidemiology Collaboration (CKD-EPI) equation [67].

### 4.4. Statistical Analysis

After checking for normality, data showing non-normal distribution were reported as the median and interquartile range (IQR), and data showing normal distribution were reported as mean with standard deviation (SD). One-way analysis of variance (ANOVA) or the Kruskal–Wallis test with post-hoc pairwise comparison based on Tukey’s test or Wilcoxon rank sum test was used for comparison among the three groups, and independent sample t-test or Wilcoxon rank sum test was used for comparison between two groups. Chi-square and Fisher’s exact tests were used to analyze categorical data. Spearman’s rank correlation was used for correlation analysis [68]. Univariate and multivariable logistic regression analyses were performed to evaluate the predictor variables for the development of VIKI including serum biomarker concentration in the validation phase and other clinical parameters. Initially, we performed the univariate logistic regression analysis with each serum biomarker concentration and the following parameters: age, sex, comorbidities (hypertension, diabetes), BMI, CRP, vancomycin trough concentration, baseline sCr, duration of vancomycin therapy, use of concomitant nephrotoxic agent, and eGFR. Subsequently, we performed the multivariable logistic regression analysis adjusted by age, sex, BMI, and CRP. The diagnostic performance of each biomarker for VIKI was assessed using the AUC in the ROC analysis [69]. A *p* value < 0.05 was considered statistically significant. Statistical analyses were performed using SAS version 9.4 (SAS Institute, Cary, NC, USA), IBM SPSS^®^ Statistics version 25 (IBM, Armonk, NY, USA), and R 4.0.3 (Vienna, Austria; http://www.R-project.org/, accessed on 24 August 2021).

## Figures and Tables

**Table 1:** Baseline subject characteristics.

	VIKI (*n* = 28)	Non-VIKI (*n* = 21)	HC (*n* = 23)	*p* Value
Age, years	64 (53–69)	61 (56–67)	61 (55–66)	0.777
Male, *n* (%)	18 (64)	12 (57)	13 (57)	0.819
BMI ^1^, kg/m^2^	21.8 ± 3.2	23.0 ± 3.5	24.6 ± 2.7	**0.011**
Overweight (BMI > 25 kg/m^2^), *n* (%)	7 (25)	6 (29)	10 (43)	0.390
Serum Creatinine, mg/dL	1.60 (1.30–2.09)	0.62 (0.49–0.71)	0.85 (0.77–0.96)	**<0.001**
eGFR, mL/min/1.73 m^2^	42 (31–52)	107 (88–121)	85 (78–93)	**<0.001**
CRP ^2^, mg/dL	5.78 (2.25–10.08)	6.31 (2.80–9.56)	0.04 (0.03–0.09)	**<0.001**
Vancomycin trough concentration ^1^, μg/mL	27.3 ± 5.3	14.3 ± 2.8		**<0.001**
Duration of vancomycin therapy, days	6 (3–9)	6 (4–11)		0.570
Concomitant nephrotoxic agent use, *n* (%)	17 (61)	11 (52)		0.560
Site of infection, *n* (%)				0.419
Respiratory	9 (32%)	7 (33%)		
Blood stream	8 (29%)	2 (10%)		
Central nervous system	3 (11%)	4 (19%)		
Others	8 (29%)	8 (38%)		

Note: *p* value < 0.05 was considered statistically significant. Significant values are indicated in bold. ^1^ All of the values were described in the median with interquartile range except for BMI and vancomycin trough concentration which were described in mean ± standard deviation. ^2^ Subjects without the result of CRP existed. Abbreviation: VIKI, vancomycin induced kidney injury; HC, healthy control; BMI, body mass index; eGFR, estimated glomerular filtration rate; CRP, C-reactive protein.

**Table 2:** Serum biomarker concentrations in the (**A**) screening test by Luminex assay (*n* = 55) and the (**B**) validation test by ELISA (*n* = 72).

(**A**)								
	**VIKI** **(*n* = 20)**	**Control**		**VIKI vs. Control**	**VIKI vs. Non-VIKI vs. HC**	**Post Hoc Comparison**		
		**Non-VIKI** **(*n* = 15)**	**HC** **(*n* = 20)**			**VIKI vs. HC**	**VIKI vs. Non-VIKI**	**Non-VIKI vs. HC**
**IL-18 (pg/mL)**	849 (468–1352)	455 (347–766)	229 (190–303)	**<0.001**	**<0.001**	**<0.001**	**0.021**	**<0.001**
**TNF-R1 (pg/mL)**	6797 (4085–11,770)	3735 (2411–4920)	1017 (918–1120)	**<0.001**	**<0.001**	**<0.001**	**0.001**	**<0.001**
**CXCL10 (pg/mL)**	563.3 (189.0–1161.3)	199.8 (139.6–305.7)	77.9 (61.7–89.3)	**<0.001**	**<0.001**	**<0.001**	**0.031**	**<0.001**
**Osteopontin (ng/mL)**	193.1 (103.2–334.0)	73.8 (42.5–109.7)	17.8 (16.1–28.1)	**<0.001**	**<0.001**	**<0.001**	**<0.001**	**<0.001**
**TFF3 (ng/mL)**	8.5 (4.8–14.9)	2.9 (1.7–4.7)	1.9 (1.6–2.2)	**<0.001**	**<0.001**	**<0.001**	**<0.001**	**0.023**
**Clusterin (μg/mL)**	0.20 (0.15–0.24)	0.19 (0.15–0.22)	0.23 (0.20–0.24)	0.489	0.073			
**Cystatin C (mg/L)**	2.7 (2.2–3.2)	1.2 (1.0–1.6)	1.2 (1.1–1.3)	**<0.001**	**<0.001**	**<0.001**	**<0.001**	0.994
**RBP4 (μg/mL)**	30.4 (23.4–41.4)	19.5 (13.2–36.2)	62.3 (51.5–82.7)	0.057	**<0.001**	**<0.001**	0.089	**<0.001**
**NGAL (ng/mL)**	130.4 (101.1–155.1)	70.5 (49.0–116.8)	55.4 (37.0–60.3)	**<0.001**	**<0.001**	**<0.001**	**0.006**	0.105
(**B**)								
	**VIKI** **(*n* = 28)**	**Control**		**VIKI vs. Control**	**VIKI vs. Non-VIKI vs. HC**	**Post Hoc Comparison**		
		**Non-VIKI** **(*n* = 21)**	**HC** **(*n* = 23)**			**VIKI vs. HC**	**VIKI vs. Non-VIKI**	**Non-VIKI vs. HC**
**IL-18 (pg/mL)**	461 (363–798)	266 (161–442)	122 (95–164)	**<0.001**	**<0.001**	**<0.001**	**0.017**	**<0.001**
**TNF-R1 (pg/mL)**	7915 (4655–12,871)	2845 (1820–4444)	1149 (975–1336)	**<0.001**	**<0.001**	**<0.001**	**0.002**	**0.005**
**CXCL10 (pg/mL)**	509.2 (184.2–748.8)	221.2 (135.4–317.5)	96.0 (66.4–125.4)	**<0.001**	**<0.001**	**<0.001**	**0.034**	**<0.001**
**Osteopontin (ng/mL)**	9.5 (5.3–14.6)	3.5 (2.3–7.2)	1.0 (0.8–1.2)	**<0.001**	**<0.001**	**<0.001**	**<0.001**	**<0.001**
**TFF3 (ng/mL)**	27.5 (16.8–39.0)	8.4 (6.07–12.4)	6.5 (5.7–8.9)	**<0.001**	**<0.001**	**<0.001**	**<0.001**	0.394
**Cystatin C (mg/L)**	2.3 (1.6–2.8)	0.8 (0.7–1.0)	0.7 (0.6–0.9)	**<0.001**	**<0.001**	**<0.001**	**<0.001**	0.071
**NGAL (ng/mL)**	157.5 (97.3–362.1)	96.9 (53.3–141.7)	38.8 (28.1–48.9)	**<0.001**	**<0.001**	**<0.001**	**0.011**	**<0.001**

Note: *p*-value < 0.05 was considered statistically significant. Significant values are indicated in bold. Abbreviations: VIKI, vancomycin-induced kidney injury; HC, healthy control; IL-18, interleukin-18; TNF-R1, tumor necrosis factor receptor 1; CXCL10, C-X-C motif chemokine ligand 10; TFF3, trefoil factor-3; RBP4, retinol binding protein 4; and NGAL, neutrophil gelatinase-associated lipocalin.

**Table 3:** Multivariable logistic regression analysis for serum biomarkers in association with the development of VIKI (*n* = 49). The regression model was adjusted for age, sex, body mass index, and CRP.

Independent Variables	Odds Ratio [95% Confidence Interval]	*p* Value
**IL-18**	1.001 (0.999–1.002)	0.285
**TNF-R1**	**1.001 (1.000–1.001)**	**0.002**
**CXCL10**	**1.003 (1.000–1.006)**	**0.024**
**OPN**	**1.000 (1.000–1.000)**	**0.024**
**TFF3**	**1.000 (1.000–1.001)**	**0.002**
**Cystatin C**	**1.003 (1.001–1.005)**	**<0.001**
**RBP4**	1.000 (1.000–1.000)	0.724
**NGAL**	**1.007 (1.001–1.013)**	**0.026**

Note: *p* value < 0.05 was considered statistically significant. Significant values are indicated in bold. Abbreviations: VIKI, vancomycin-induced kidney injury; CRP, C-reactive protein; IL-18, interleukin-18; TNF-R1, tumor necrosis factor receptor 1; CXCL10, C-X-C motif chemokine ligand 10; TFF3, trefoil factor-3; RBP4, retinol binding protein 4; NGAL, neutrophil gelatinase-associated lipocalin; BMI, body mass index.

## Supplementary Materials

The following are available online at https://www.mdpi.com/2077-0383/10/21/5005/s1. Figure S1: Correlation analyses and scatter plots between serum biomarker concentrations and estimated glomerular filtration rate in the screening test (*n* = 55). Table S1: Univariate logistic regression analysis for serum biomarkers and clinical parameters in association with the development of VIKI.
