# Correction: Kim et al. Diagnostic Value of Multiple Serum Biomarkers for Vancomycin-Induced Kidney Injury. *J. Clin. Med.* 2021, *10*, 5005

**DOI:** 10.3390/jcm11061667

**Published:** 2022-03-17

**Authors:** Sang-Mi Kim, Hyun-Seung Lee, Min-Ji Kim, Hyung-Doo Park, Soo-Youn Lee

**Affiliations:** 1 Samsung Medical Center, Department of Laboratory Medicine and Genetics, School of Medicine, Sungkyunkwan University, Seoul 06351, Korea; jeehee0520@gmail.com (S.-M.K.); hyunseung1011.lee@samsung.com (H.-S.L.); nayadoo@hanmail.net (H.-D.P.); 2 Biomedical Statistics Center, Research Institute for Future Medicine, Samsung Medical Center, Seoul 06351, Korea; rabbit93.kim@samsung.com; 3 Samsung Medical Center, Department of Clinical Pharmacology & Therapeutics, School of Medicine, Sungkyunkwan University, Seoul 06351, Korea; 4 Department of Health Science and Technology, Samsung Advanced Institute of Health Science and Technology, Sungkyunkwan University, Seoul 06351, Korea

**Error in Table**

In the original publication [1], there was a mistake in “***Table 2. Serum biomarker concentrations in the (A) screening test by Luminex assay (n = 55) and the (B) validation test by ELISA (n = 72)***.” as published. ** Table 2(B) was identical to Table 2(A)**. The corrected “***Table 2. Serum biomarker concentrations in the (A) screening test by Luminex assay (n = 55) and the (B) validation test by ELISA (n = 72).***” appears below.

**Text Correction**

There was an error in the original publication. **A word needs to be replaced in the sentence**.

A correction has been made to **3. Discussion**, **Paragraph 8**:

**There are some limitations that must be noted. First, owing to the retrospective nature of our study, the study evaluated the diagnostic value of the biomarkers rather than their predictive value for VIKI. Secondly, this study identified candidate serum biomarkers that have potential for the diagnosis of VIKI, but the current design could not show their specificity for VIKI. Indeed, we are conducting an additional experiment with a control group consisting of patients with AKI induced by causes other than vancomycin to identify the specificity of the biomarkers for VIKI. Lastly, this research was a single-center study with a limited number of subjects with heterogeneous baseline conditions, preventing our results from being free of bias. Large-scale, multi-center studies would be required to establish the credibility of our findings.**

The authors apologize for any inconvenience caused and state that the scientific conclusions are unaffected. This correction was approved by the Academic Editor. The original publication has also been updated.

## Figures and Tables

**Table 2:** Serum biomarker concentrations in the (**A**) screening test by Luminex assay (*n* = 55) and the (**B**) validation test by ELISA (*n* = 72).

(**A**)								
	**VIKI** **(*n* = 20)**	**Control**		**VIKI vs. Control**	**VIKI vs. Non-VIKI vs. HC**	**Post Hoc Comparison**		
		**Non-VIKI** **(*n* = 15)**	**HC** **(*n* = 20)**			**VIKI vs. HC**	**VIKI vs. Non-VIKI**	**Non-VIKI vs. HC**
**IL-18 (pg/mL)**	849 (468–1352)	455 (347–766)	229 (190–303)	**<0.001**	**<0.001**	**<0.001**	**0.021**	**<0.001**
**TNF-R1 (pg/mL)**	6797 (4085–11,770)	3735 (2411–4920)	1017 (918–1120)	**<0.001**	**<0.001**	**<0.001**	**0.001**	**<0.001**
**CXCL10 (pg/mL)**	563.3 (189.0–1161.3)	199.8 (139.6–305.7)	77.9 (61.7–89.3)	**<0.001**	**<0.001**	**<0.001**	**0.031**	**<0.001**
**Osteopontin (ng/mL)**	193.1 (103.2–334.0)	73.8 (42.5–109.7)	17.8 (16.1–28.1)	**<0.001**	**<0.001**	**<0.001**	**<0.001**	**<0.001**
**TFF3 (ng/mL)**	8.5 (4.8–14.9)	2.9 (1.7–4.7)	1.9 (1.6–2.2)	**<0.001**	**<0.001**	**<0.001**	**<0.001**	**0.023**
**Clusterin (μg/mL)**	0.20 (0.15–0.24)	0.19 (0.15–0.22)	0.23 (0.20–0.24)	0.489	0.073			
**Cystatin C (mg/L)**	2.7 (2.2–3.2)	1.2 (1.0–1.6)	1.2 (1.1–1.3)	**<0.001**	**<0.001**	**<0.001**	**<0.001**	0.994
**RBP4 (μg/mL)**	30.4 (23.4–41.4)	19.5 (13.2–36.2)	62.3 (51.5–82.7)	0.057	**<0.001**	**<0.001**	0.089	**<0.001**
**NGAL (ng/mL)**	130.4 (101.1–155.1)	70.5 (49.0–116.8)	55.4 (37.0–60.3)	**<0.001**	**<0.001**	**<0.001**	**0.006**	0.105
(**B**)								
	**VIKI** **(*n* = 28)**	**Control**		**VIKI vs. Control**	**VIKI vs. Non-VIKI vs. HC**	**Post Hoc Comparison**		
		**Non-VIKI** **(*n* = 21)**	**HC** **(*n* = 23)**			**VIKI vs. HC**	**VIKI vs. Non-VIKI**	**Non-VIKI vs. HC**
**IL-18 (pg/mL)**	461 (363–798)	266 (161–442)	122 (95–164)	**<0.001**	**<0.001**	**<0.001**	**0.017**	**<0.001**
**TNF-R1 (pg/mL)**	7915 (4655–12,871)	2845 (1820–4444)	1149 (975–1336)	**<0.001**	**<0.001**	**<0.001**	**0.002**	**0.005**
**CXCL10 (pg/mL)**	509.2 (184.2–748.8)	221.2 (135.4–317.5)	96.0 (66.4–125.4)	**<0.001**	**<0.001**	**<0.001**	**0.034**	**<0.001**
**Osteopontin (ng/mL)**	9.5 (5.3–14.6)	3.5 (2.3–7.2)	1.0 (0.8–1.2)	**<0.001**	**<0.001**	**<0.001**	**<0.001**	**<0.001**
**TFF3 (ng/mL)**	27.5 (16.8–39.0)	8.4 (6.07–12.4)	6.5 (5.7–8.9)	**<0.001**	**<0.001**	**<0.001**	**<0.001**	0.394
**Cystatin C (mg/L)**	2.3 (1.6–2.8)	0.8 (0.7–1.0)	0.7 (0.6–0.9)	**<0.001**	**<0.001**	**<0.001**	**<0.001**	0.071
**NGAL (ng/mL)**	157.5 (97.3–362.1)	96.9 (53.3–141.7)	38.8 (28.1–48.9)	**<0.001**	**<0.001**	**<0.001**	**0.011**	**<0.001**

Note: *p*-value < 0.05 was considered statistically significant. Significant values are indicated in bold. Abbreviations: VIKI, vancomycin-induced kidney injury; HC, healthy control; IL-18, interleukin-18; TNF-R1, tumor necrosis factor receptor 1; CXCL10, C-X-C motif chemokine ligand 10; TFF3, trefoil factor-3; RBP4, retinol binding protein 4; and NGAL, neutrophil gelatinase-associated lipocalin.
