# Supplementary material for: Diagnostic Value of Multiple Serum Biomarkers for Vancomycin-Induced Kidney Injury
Source: J Clin Med. 2021 Oct 27;10(21):5005. doi: 10.3390/jcm10215005 (PMC8584616; doi:10.3390/jcm10215005)
Supplement: Supplementary file 1 [file jcm-10-05005-s001.zip › jcm-1393394-supplementary.pdf]

Journal of
Clinical Medicine

MDPI

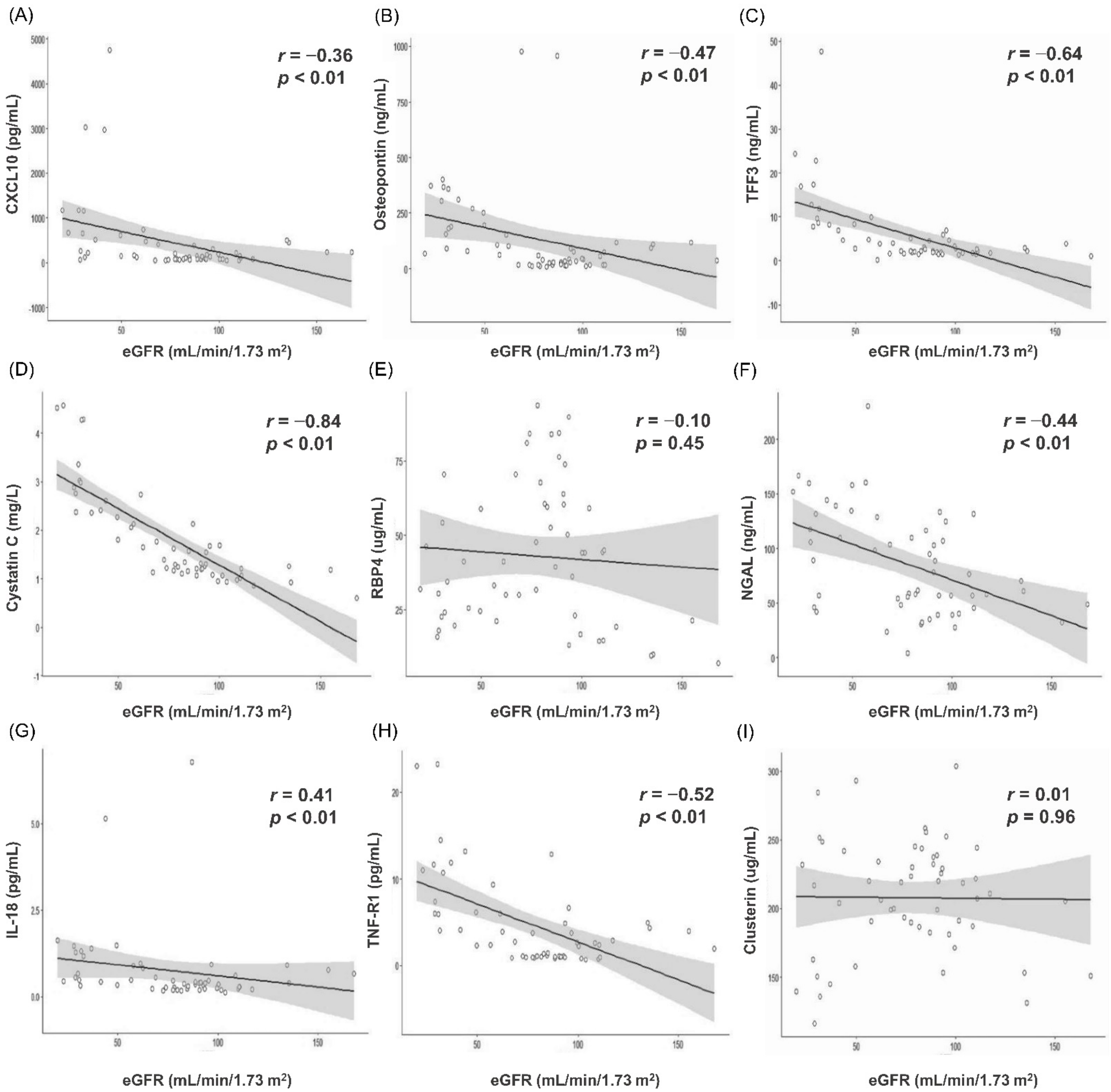

Figure S1. Correlation analyses and scatter plots between serum biomarker concentrations and estimated glomerular filtration rate in the screening test (n=55). (**A**) CXCL10 (**B**) Osteopontin (**C**) TFF3 (**D**) Cystatin C (**E**) RBP4 (**F**) NGAL (**G**) IL-18 (**H**) TNF-R1 (**I**) Clusterin. In the scatter plot, the solid line and gray zone represent the regression line and its 95% confidence interval, respectively. The value of r represents the correlation coefficient by Spearman's rho method. Abbreviation: eGFR, estimated glomerular filtration rate; CXCL10, C-X-C motif chemokine ligand 10; TFF3, trefoil factor-3; RBP4, retinol binding protein 4; NGAL, neutrophil gelatinase-associated lipocalin; IL-18, interleukin-18; TNF-R1, tumor necrosis factor receptor 1

Table S1. Univariate logistic regression analysis for serum biomarkers and clinical parameters in association with the development of VIKI.

Independent Variables	Odds Ratio (95% Confidence interval)	*P* Value
**Age**	1.010 (0.969-1.053)	0.645
**Sex**	0.741 (0.232-2.362)	0.612
**BMI**	0.897 (0.754-1.068)	0.223
**Hypertension**	0.800 (0.236-2.718)	0.721
**Diabetes**	0.873 (0.226-3.368)	0.843
**CRP**	1.033 (0.953-1.119)	0.428
**Vancomycin trough concentration**	<0.001 (0.000-0.000)	0.994
**Baseline sCr**	1.961 (0.186-20.724)	0.576
**eGFR**	0.019 (0.000-0.000)	0.994
**Duration of vancomycin therapy**	0.964 (0.904-1.028)	0.260
**Use of concomitant nephrotoxic agent**	1.405 (0.448-4.410)	0.560
**IL-18**	1.001 (0.999-1.002)	0.279
**TNF-R1**	1.000 (1.000-1.001)	0.003
**CXCL10**	**1.003 (1.001-1.006)**	**0.017**
**OPN**	1.000 (1.000-1-000)	0.057
**TFF3**	1.000 (1.000-1-000)	0.002
**Cystatin C**	**1.003 (1.001-1.004)**	**<0.001**
**RBP4**	1.000 (1.000-1.000)	0.783
**NGAL**	**1.007 (1.001-1.013)**	**0.016**

Note: *P* value < 0.05 was considered statistically significant. Significant values are indicated in bold.

Abbreviation: VIKI, vancomycin-induced kidney injury; BMI, body mass index; CRP, C-reactive protein; eGFR, estimated glomerular filtration rate; sCr, serum creatinine; IL-18, interleukin-18; TNF-R1, tumor necrosis factor receptor 1; CXCL10, C-X-C motif chemokine ligand 10; TFF3, trefoil factor-3; RBP4, retinol binding protein 4; NGAL, neutrophil gelatinase-associated lipocalin.
